# Combined with UPLC-Triple-TOF/MS-based plasma lipidomics and molecular pharmacology reveals the mechanisms of schisandrin against Alzheimer’s disease

**DOI:** 10.1186/s13020-023-00714-y

**Published:** 2023-02-06

**Authors:** Tian-tian Zhao, Ying Zhang, Cheng-qin Zhang, Ya-fei Chang, Mei-rong Cui, Yue Sun, Wen-qian Hao, Yu-meng Yan, Shuo Gu, Yao Xie, Bin-bin Wei

**Affiliations:** 1 Central Laboratory, School of Pharmacy, China Medical University, No.77 Puhe Road, Shenyang, 110122 People’s Republic of China; 2 Hubei Three Gorges Polytechnic, No.31 Stadium Road, Yichang, 443000 People’s Republic of China

**Keywords:** Alzheimer’s disease, Schisandrin, Lipidomics, BV2 microglia, LXR, APOE

## Abstract

**Background:**

Alzheimer’s disease (AD), a type of neurodegeneration disease, is characterized by Aβ deposition and tangles of nerve fibers. Schisandrin is one of the main components of *Fructus Schisandrae Chinensis*. Researches showed that schisandrin can improve the cognitive impairment and memory of AD mice, but the specific mechanism has not been fully elucidated.

**Purpose:**

The purpose of this study is to investigate the possible mechanism of schisandrin in improving AD pathology.

**Methods:**

The Morris water maze test was executed to detect spatial learning and memory. Ultra performance liquid chromatography-Triple time of flight mass spectrometry (UPLC-Triple-TOF/MS)-based plasma lipidomics was used to study the changes of plasma lipids. Moreover, we measured the levels of protein and mRNA expression of APOE and ABCA1 in the rat brains and in BV2 microglia.

**Results:**

Our study found that schisandrin could improve learning and memory, and reduce Aβ deposition in AD rats. Furthermore, we found that schisandrin can improve plasma lipid metabolism disorders. Therefore, we hypothesized schisandrin might act via LXR and the docking results showed that schisandrin interacts with LXRβ. Further, we found schisandrin increased the protein and mRNA expression of LXR target genes APOE and ABCA1 in the brain of AD rats and in BV2 microglia.

**Conclusion:**

Our study reveals the neuroprotective effect and mechanism of schisandrin improves AD pathology by activating LXR to produce APOE and ABCA1.

**Supplementary Information:**

The online version contains supplementary material available at 10.1186/s13020-023-00714-y.

## Introduction

Alzheimer’s disease (AD), the most common cause of dementia, is a growing global health concern with huge implications for individuals and society [12]. AD is a complex and progressive neurodegenerative disease with many pathogenic factors [19]. The main pathological features of AD are deposition of amyloid (Aβ) and tangles of phosphorylated Tau protein [18]. The early pathological markers of AD are the formation of soluble Aβ oligomer and synaptic dysfunction [11]. There is a dynamic equilibrium between the production and clearance of Aβ. In addition, many studies have shown that neuroinflammation is involved in the pathogenesis of AD [4]. Microglia are innate immune cells in the brain that are central mediators of neuroinflammatory responses [29]. Thus, AD is associated with microglia activation.

The brain is one of the most lipid rich organs, so lipidomics may provide important information about the role of lipids in AD, and many studies have shown various lipid metabolism disorders in the early stage of AD [25]. Liver X Receptor (LXR), as lipid-activated transcription factors in the nuclear receptor superfamily [23], is a key regulator of cholesterol homeostasis and inflammatory signals in the central nervous system [28]. Apolipoprotein E (APOE) and ATP binding cassette transporter A1 (ABCA1) are LXR target genes. As an important component of plasma lipoprotein, APOE is involved in lipid transport [30], especially the transport of cholesterol. In brain tissues, APOE is secreted by glial cells and is lipidized by ABCA1 and ATP-binding cassette G1(ABCG1), and the increased secretion of APOE lipoprotein can promote the clearance of Aβ [13]. In addition, LXR regulates microglia activation and neuroinflammatory response [29].

*Schisandra chinensis*, as a medicinal and food homologous plant, has very important medicinal and edible value, and its main ingredient is schisandrin. Schisandrin is a lignan class of compounds that act on nerve cells across the Blood–Brain Barrier (BBB) [16]. In addition, schisandrin has anti-inflammatory [3] and pharmacological effects on improving learning and memory ability [9]. However, the mechanism by which schisandrin acts on AD has not been fully elucidated.

Since schisandrin has a good ability to penetrate the BBB, we speculate that schisandrin may function by acting on the brain. Because changes in the brain can be reflected in peripheral plasma, we speculate on the targets that schisandrin might play based on the results of plasma lipidomics. This study looks for and validates new targets from plasma lipidomics results, which provides a new idea for the treatment of AD. Therefore, our study aimed to explore the mechanism of schisandrin on AD and provide help for clinical application.

## Methods

### Animals and experimental design

**Fig. 1:**
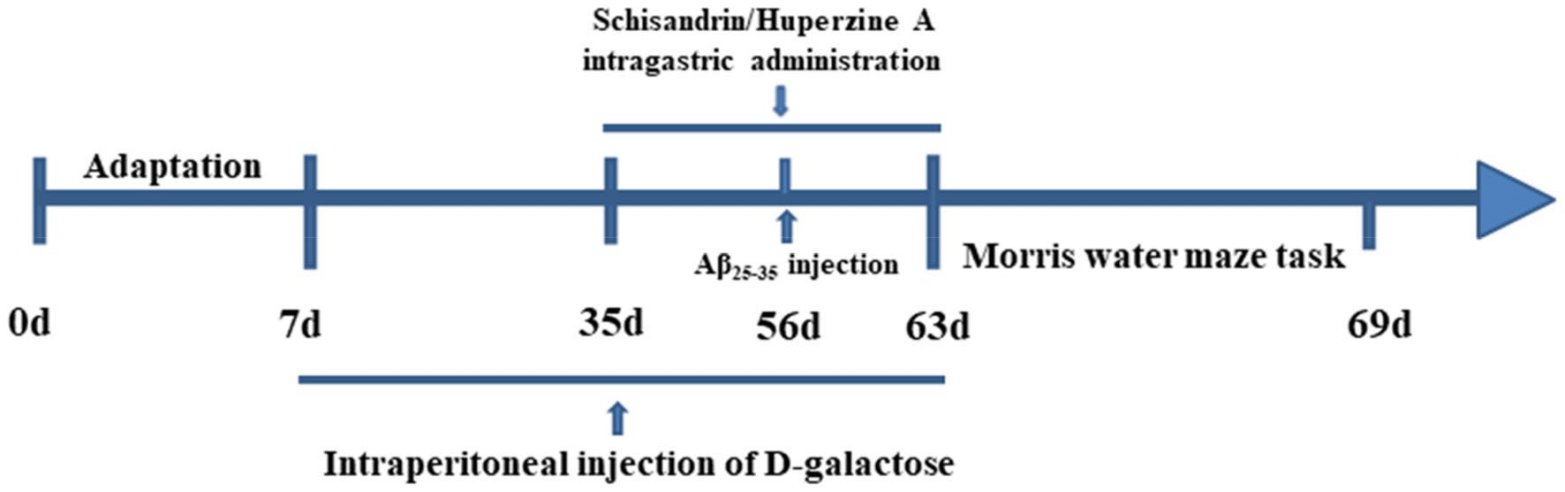
Flow chart of animal Experiments

40 SPF level SD rats weighing 200 ± 20 g were purchased from Liaoning Changsheng Biotechnology Co.,LTD. All of them were adult males. Rats were breed in an environment where temperature maintained 23 ± 2 ℃ under a 12 h light–dark cycle with a humidity of 50 ± 10%. All of the experiment were based on Animal Welfares and Ethics of China Medical University (CMU2020248). After a week of adaptation feeding, rats were divided into four groups: Control group, Model group, Schisandrin group (Sch), Huperzine A group (HupA), 10 rats in each group. Model, Sch and HupA groups were treated with D-galactose and Aβ_25-35_ [5, 7, 27]. Model, Sch and HupA groups were intraperitoneally injected with D-galactose 120 mg/kg/d for eight weeks. From the 5th week, Sch group was treated schisandrin 10 mg/kg/d intragastric, HupA group was treated Huperzine A 30 μg/kg/d intragastric, and Control group was treated with same dosage of normal saline. At the 8th week, the fasted rats were anesthetized with 0.5 g/mL uratan and fixed on the stereolocator. The scalp was cut open and the periosteum removed with a scalpel to fully expose the skull. 10 μg Aβ_25-35_ was given to each side. Control group was given a same dose of PBS (Fig. 1).

### Morris water maze (MWM) test

The MWM test was conducted at week 9. The pool was divided into 4 quadrants: qaudrant I, II, III, IV. A platform was set in the quadrant I 2–3 cm above the water surface. In positioning navigation training, the rats were put into water facing the wall at a fixed position of the four quadrants every day, record the time and trajectory of the rats searching for the platform. If the platform was not found after 60 s, the escape latency was recorded as 60 s and the experimenter guided it to find the platform. After five days of continuous training, the platform was removed, and the rats were placed in the water maze from the quadrant III furthest from the platform. The swimming track, the times of crossing the platform and the time of staying in the target quadrant were recorded.

### Immunohistochemistry

Slices are routinely dewaxed into water; Sodium citrate repair solution high pressure repair for 10 min; PBS wash 3 times for 3 min each; Peroxidase removal of 3% H_2_O_2_; Goat serum was blocked for 30 min; Primary antibody incubation (overnight at 4 °C); PBS wash 3 times for 3 min each; Secondary antibody incubation for 30 min; PBS wash 3 times for 5 min each; DAB color rendering; Hematoxylin dye solution 5 min; Conventional dehydration transparent; Gum seals.

### Lipid extraction

The blood samples added with EDTA were centrifuged at 4000 rpm at 4 ℃ for 15 min to obtain plasma. 150 μL methanol was added to the 100 μL plasma sample and then vorticised for 5 s. The following was to add 750 μL MTBE, place 30 min at room temperature, then add 225 μL water, scroll for 20 s and place 10 min at room temperature. The solution was centrifuged at 15,000 rpm at 4 ℃ for 10 min and stratified. The upper layer was shifted to a new tube, desiccated under vacuum. Then, the dried samples were reconstituted with MeOH/DCM (100 μL, 1:1, v/ v) and treated by ultrasound for 5 min. At last, the solution was centrifuged at 12,000 rpm at 4 ℃ for 15 min, and the supernatant was taken into the sample bottle for sample analysis. Quality control (QC) samples were obtained by pooling and blending equal aliquots of each sample and detected.

### LC–MS Condition for lipid analysis

ACQUITY HSS T3 column (2.1 × 100 mm, 1.8 μm, Waters); Column temperature: 40 ℃; The flow rate of 0.3 mL/min; The injection volume was 2 μL; Mobile phase A is methanol: acetonitrile: water (1:1:1, containing 5 mM ammonium acetate); Mobile phase B: Isopropyl alcohol (containing 5 mM ammonium acetate); Mobile Phase constitution was changed as followed: 5–95% B at 0–18 min, 95% B at 18–21 min, 5% B at 21.1–25 min. Mass spectrometry was acquired on a Triple TOF^®^ 5600 System (AB SCIEX, USA) in positive and negative ion modes. TOF MS was followed by 10 product ion scan with accumulation time of 50 millsecond per MS/MS and the mass range was 50 to 1250 m/z. The source conditions were set as follows: spray voltage (ISVF) of 5500 V in ESI ( +) and − 4500 V in ESI (−), collision energy: 40 V ( +) and − 40 V (−); collision energy spread: 20 V; declustering potential: 80 V ( +) and − 80 V (−), nebulizing gas (GS1) at 50 psi, heating gas (GS2) at 55 psi, curtain gas (CUR) at 35 psi, and heater temperature (TEM) at 600 °C.

### Data processing

The acquired, corrected LC–MS data were imported to Progensis QI group by group for peak picking, alignment and normalization. The adduct ion forms, involving [M + H]^+^, [M + Na]^+^, [M + NH4]^+^ in the positive ion mode and [M−H]^−^, [M + FA−H]^−^ in the negative ion mode were selected based on the ionization behaviours. In the identification analysis, lipid metabolites were identified by their m/z, mass spectra, retention time and characteristic ion fragments and further by searching the Human Metabolome Database (HMDB) (http://www.hmdb.ca/), LIPIDMAPS(http://www.lipidmaps.org/) and KEGG (http://www.genome.jp/kegg/) databases. The obtained data were input into EZinfo software for unsupervised principal component analysis (PCA) and supervised orthogonal partial least squares discriminant analysis (OPLS-DA). QC can fully ensure the accuracy and repeatability of experimental results.

### Molecular docking

Molecular docking study was carried out using Molecular Operating Environment. The protein molecule included in our study, LXRβ was obtained from Protein Data Bank [PDB Code 1PQC]. The best conformation was selected according to molecular docking score and interaction.

### CCK8 assays BV2 cell viability

The concentration setting of Aβ model drug: 1 μM, 5 μM, 10 μM, 20 μM, 40 μM, the concentration setting of schisandrin: 0.5 μM, 1 μM, 2.5 μM, 5 μM, 10 μM. TO901317 dose concentration reference study, the dose time was set at 24 h [14, 26]. Each group has 6 compound holes. Data analysis was performed according to OD values, and the experiment was repeated 3 times.

### Annexin V-FITC/PI dual staining for BV2 cell apoptosis

BV2 cells were induced by apoptosis according to the experimental scheme, centrifuged at 300*g* for 5 min, supernatant was discarded, cells were collected, washed by PBS once, cells were gently suspended and counted. 1–5 × 10^5^ suspended cells were centrifuged at 300*g* for 5 min, and the supernatant was discarded. The cells were washed with PBS once, the supernatant was discarded after centrifugation, and the cells were re-suspended with 100 μL diluted 1 × Annexin V Binding Buffer. 2.5 μL Annexin V-FITC and 2.5 μL PI staining solution were added to cell suspension. After mixing with gentle vorticity, incubate for 15–20 min at room temperature away from light. 400 μL diluted 1 × Annexin V Binding Buffer was added, and the samples were mixed. Check on the machine immediately.

### Western blot

Brain tissue was lysed using a strong protein lysate RIPA supplemented with a protease inhibitor and homogenized at 4 °C. The homogenate was centrifuged at 12,000*g* for 15 min at 4 °C, and the supernatant was taken. The collected cells are lysed with a weak protein lysate RIPA with protease inhibitors added. Protein concentration was measured using BCA reagent. Primary antibodies were incubated overnight at 4 °C. Immunoreactive bands were detected using an enhanced chemiluminescence kit and quantified with a gel-image analyzing system. The optical density of the target band was analyzed by ImageJ software processing system.

### Elisa

9 mg brain tissue was accurately weighed and 100 mL PBS was added and homogenized. After centrifugation (4 ℃, 12,000*g*, 20 min), the supernatant was collected as the sample to be tested. Collect the cell supernatant in the dish, centrifuge (4 ℃, 12,000*g*, 20 min), and collect the supernatant carefully. Collect the cells and dilute the cell suspension with PBS at a cell concentration of around 1 million/ml. Centrifuge (4 ℃, 12,000*g*, 20 min) and collect the supernatant carefully. Follow the instructions of the kit for follow-up experiments. Measure the OD value of each well at 450 nm.

### Real-time PCR

Total rat brain tissue RNA and cellular RNA were extracted with Trizol reagent, respectively, and the RNA concentration was further determined by a microplate reader. cDNA was obtained after reverse transcription. The following primers are used for brain tissue sample qPCR:

APOE-F: 5ʹ-AGGAGCAGACCCAGCAGATACG-3ʹ;

APOE-R: 5ʹ-GCAATGGAGTTGGTAGCCACAGAG-3ʹ;

ABCA1-F: 5ʹ-CACAAGCCGCCTGACTGATAAGAG-3ʹ;

ABCA1-R: 5ʹ-AGACAAGCCAATCTCCAAGCCAATC-3ʹ;

The following primers are used for cell sample qPCR:

APOE-F: 5ʹ-GAGGAACAGACCCAGCAAATA-3ʹ;

APOE-R: 5ʹ-CGATGCATGTCTTCCACTATTG-3ʹ;

ABCA1-F: 5'-CCTCAGAGAAAACAGAAAACCG-3ʹ;

ABCA1-R: 5ʹ-CTTTGCTATGATCTGCACGTAC-3ʹ;

PCR results were normalized to GAPDH mRNA levels.

### Statistical analysis

Statistical analysis was performed using GraphPad Prism 8. All data were expressed as mean and standard deviation. The comparison between the means of multiple groups of samples was performed by one-way ANOVA.

## Results

### Schisandrin improves learning and memory impairment and reduces Aβ deposition in AD rats

**Fig. 2:**
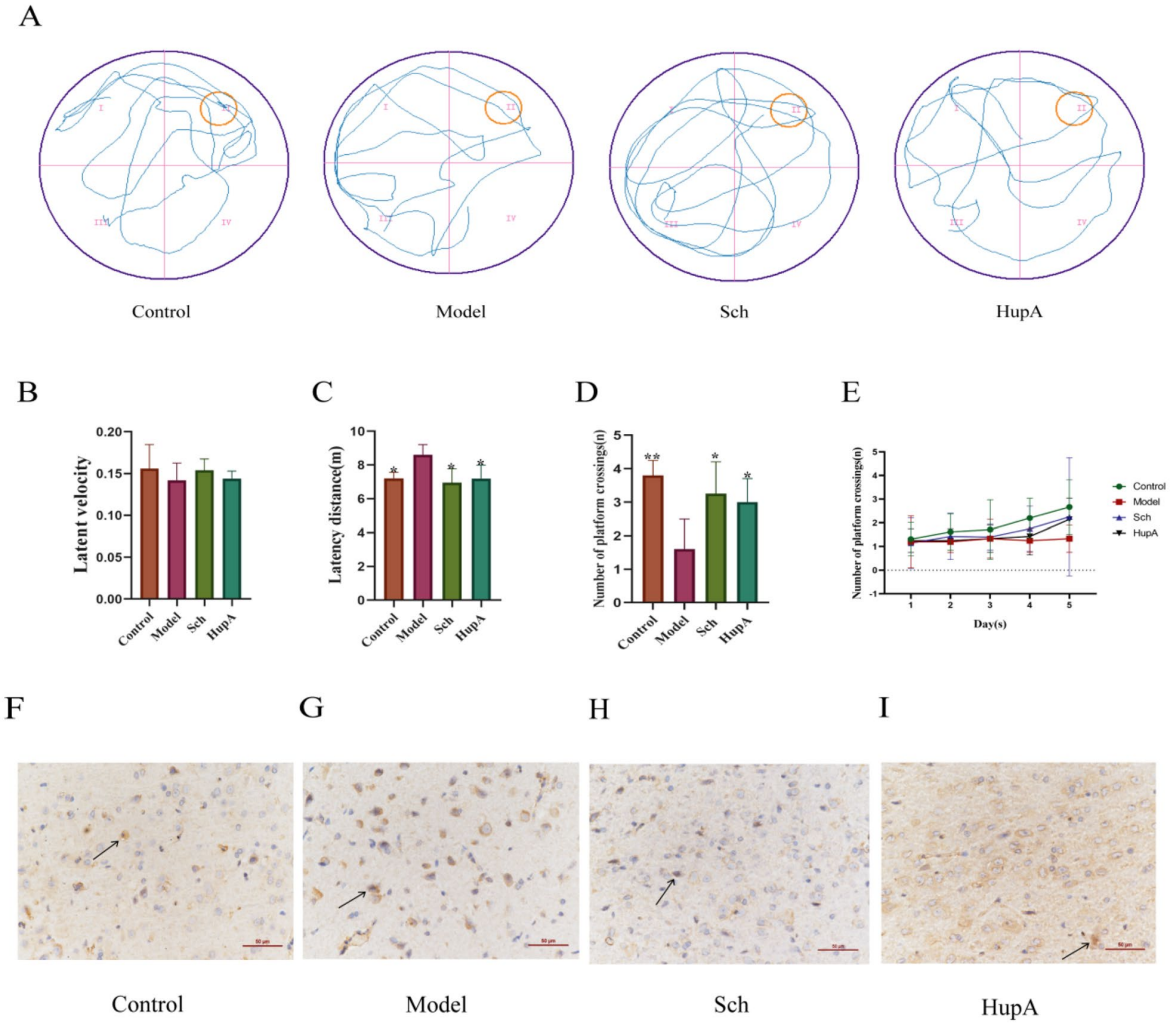
**A** the swimming track of rats; **B** the latency speed of rats; **C** the latency distance of rats; **D** the number of rats crossing the platform; **E** the number of rats crossing the platform during training. **F–I** Aβ immunohistochemistry in different groups. *represents a comparison with the Model group (*p < 0.05, **p < 0.01)

The effects of schisandrin on spatial memory ability of rats were investigated by MWM test. During training (Fig. 2B), the latency speed of each group is basically the same, indicating that the latency distance of each group has nothing to do with factors other than learning and memory. However, Fig. 2C shows that the latency distance of Model group is longer than that of Control, Sch and HupA groups, which indicates that the Model is successfully constructed and schisandrin can improve the memory ability of AD rats. In the space exploration experiment (Fig. 2D), the number of Model group crossing the platform decreased significantly, while the number of Sch and HupA groups increased but was lower than that of Control group. With the increase of training days (Fig. 2E), the shuttle times in the target quadrant of Model group remained basically unchanged, while that of Sch and HupA groups increased but were still lower than that of Control group, indicating that schisandrin can improve the learning ability of AD rats. In Fig. 2F–I, immunohistochemistry results showed that Aβ was higher in the Model group, but decreased significantly in the Sch and HupA groups. This shows that schisandrin reduces the deposition of Aβ in the brain of AD rats. In summary, our results suggest that schisandrin improves learning and memory impairment and reduces Aβ deposition in AD rats.

### Schisandrin ameliorates plasma lipid metabolism disorders

**Fig. 3:**
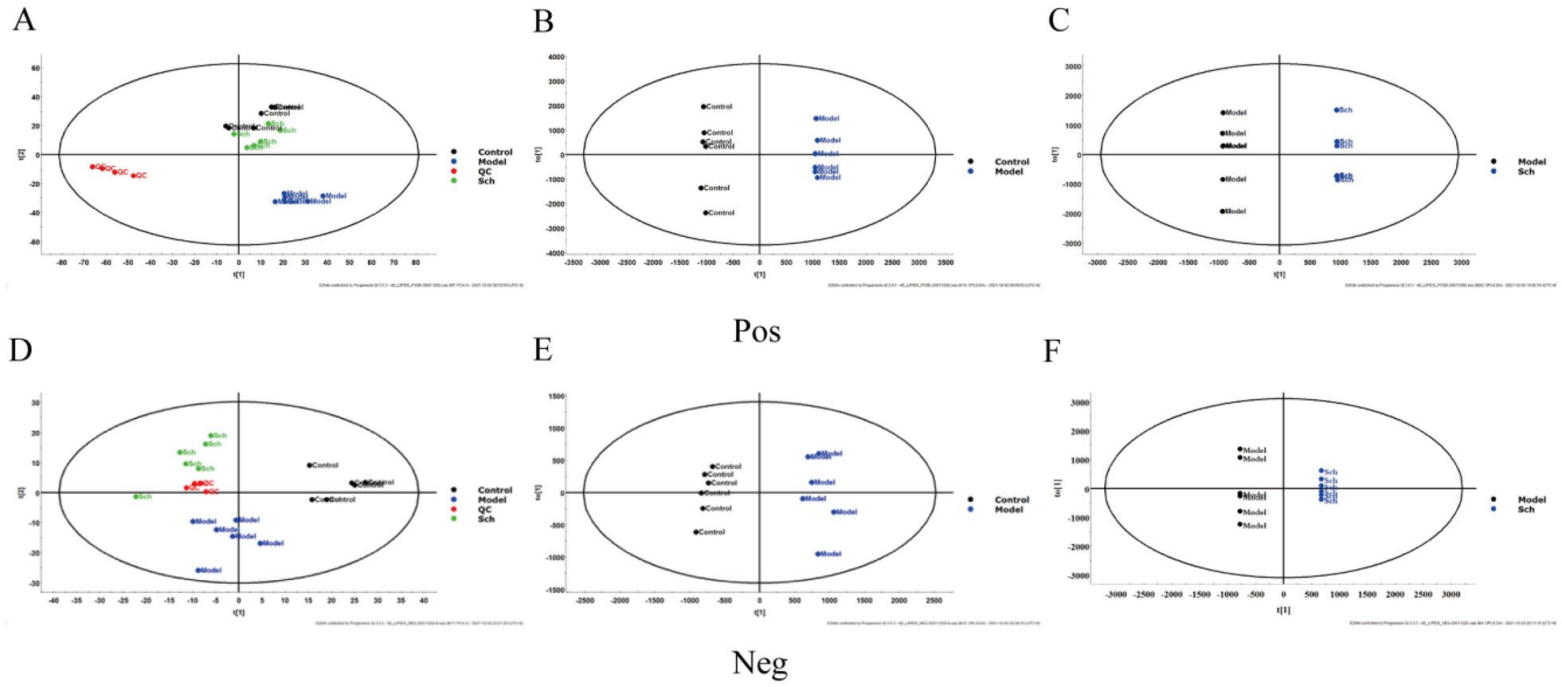
**A**, **B**, and **C** are in positive ion mode; **A** PCA score plot (R2X = 0.517 Q2 = 0.226); **B** OPLS-DA of Control and Model group (R^2^Y = 0.998 Q^2^ = 0.851); **C** OPLS-DA of Model and Sch group (R^2^Y = 0.999 Q^2^ = 0.881). **D**, **E**, and **F** are in negative ion mode; **D** PCA score plot (R^2^X = 0.454 Q^2^ = 0.360); **E** OPLS-DA of Control and Model group (R^2^Y = 0.980 Q^2^ = 0.759); **F** OPLS-DA of Model and Sch group (R^2^Y = 0.998 Q^2^ = 0.716)

Lipid differential metabolites in each group were extracted according to the conditions that CV (Variable Coefficient) ≤ 30%, ANOVA (p) < 0.05, VIP ≥ 1.0 at any age. In Fig. 3A and D, the PCA scores plot showed significant differences among the three groups. The results of PCA in the study demonstrated that schisandrin might affect plasma lipidomics in rats. To further elucidate the differential variables between the groups, OPLS-DA analysis was carried out for supervised pattern recognition. The OPLS-DA results showed that the separation of different treatment groups was obvious, indicating that lipids in the samples were significantly changed in different groups (Fig. 3B, C, E, and F). The S-plots revealed a variety of metabolites (Additional file 1: Fig. S1).

### Schisandrin may affect lipid metabolism pathways

**Fig. 4:**
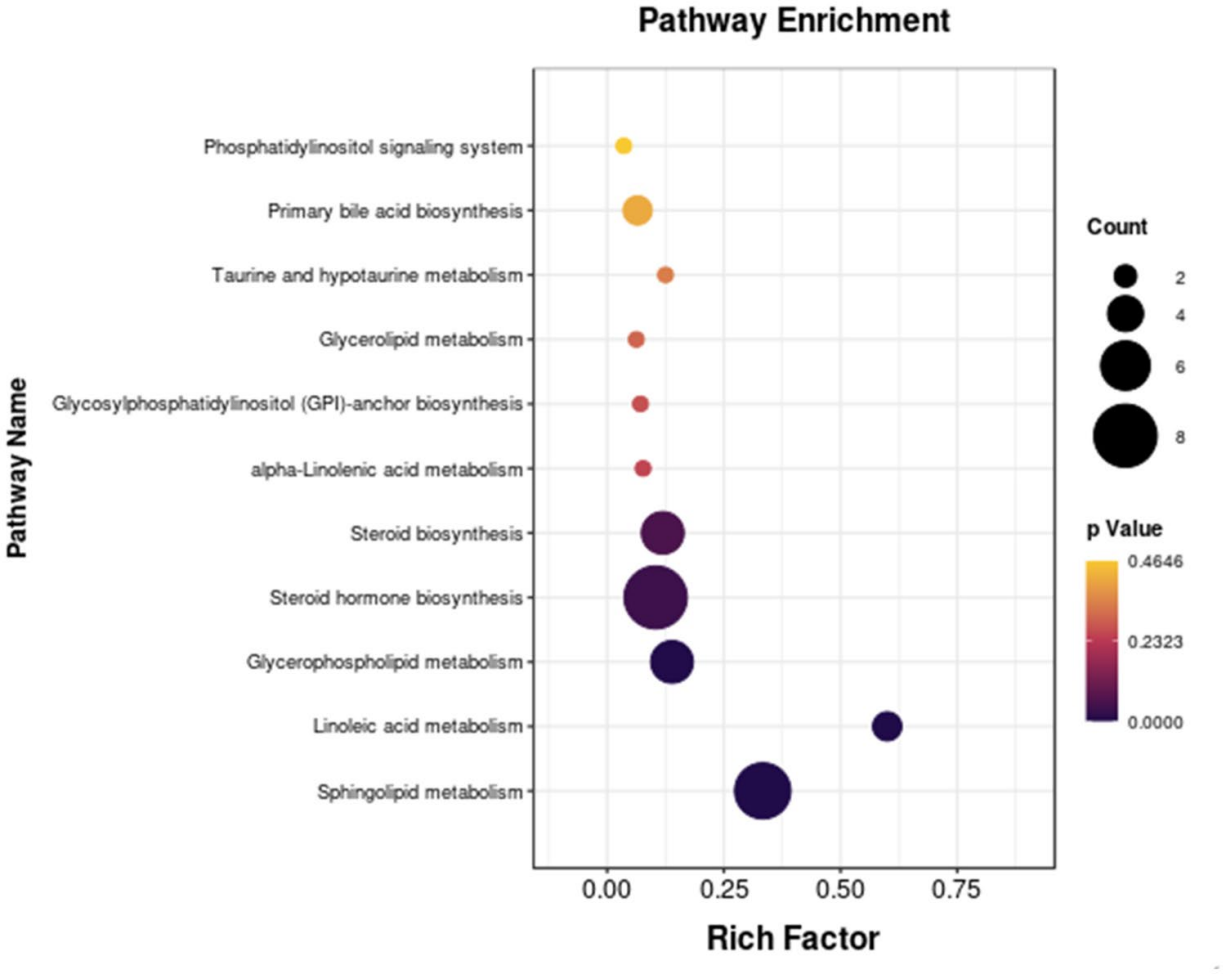
The size of the bubble represents the number of metabolites and the color of the bubble is related to the P value

MetaboAnalyst 4.0 was performed for pathway analysis to visualize the affected metabolic pathways in AD rats. As shown in Fig. 4, there are 11 metabolic pathways, but Sphingolipid metabolism, Steroid hormone biosynthesis, Steroid biosynthesis, Linoleic acid metabolism, Glycerophospholipid metabolism were considered as the crucial signaling pathways in AD and schisandrin treatment. In addition, Steroid hormone biosynthesis and Steroid biosynthesis were related to cholesterol homeostasis.

### Schisandrin may affect differential metabolite analysis

**Fig. 5:**
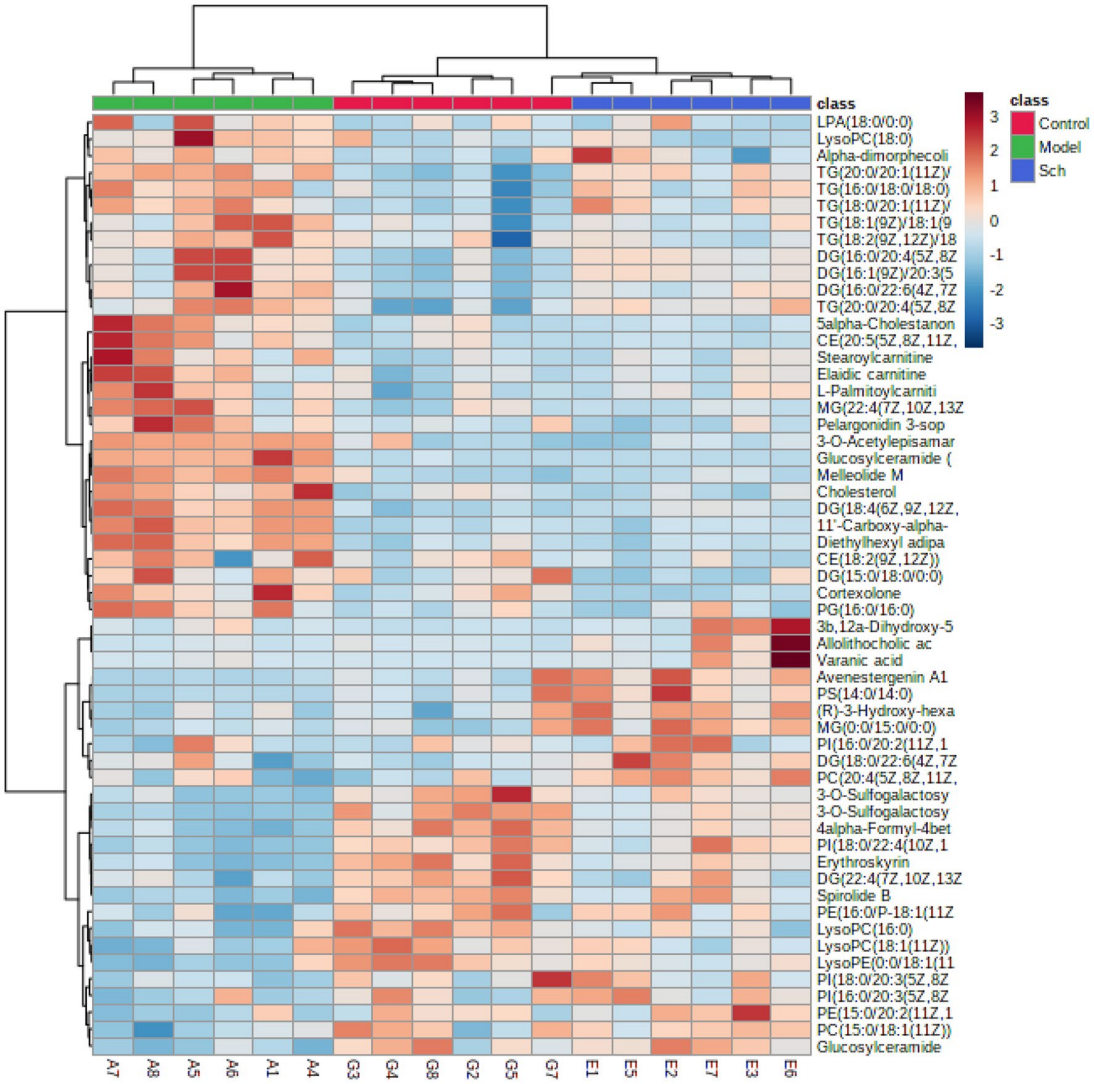
The hierarchical cluster analysis of differential metabolites in different treatment groups. Heatmap is generated in the MetaboAnalyst 4.0 (http://www.metaboanalyst.ca/)

**Fig. 6:**
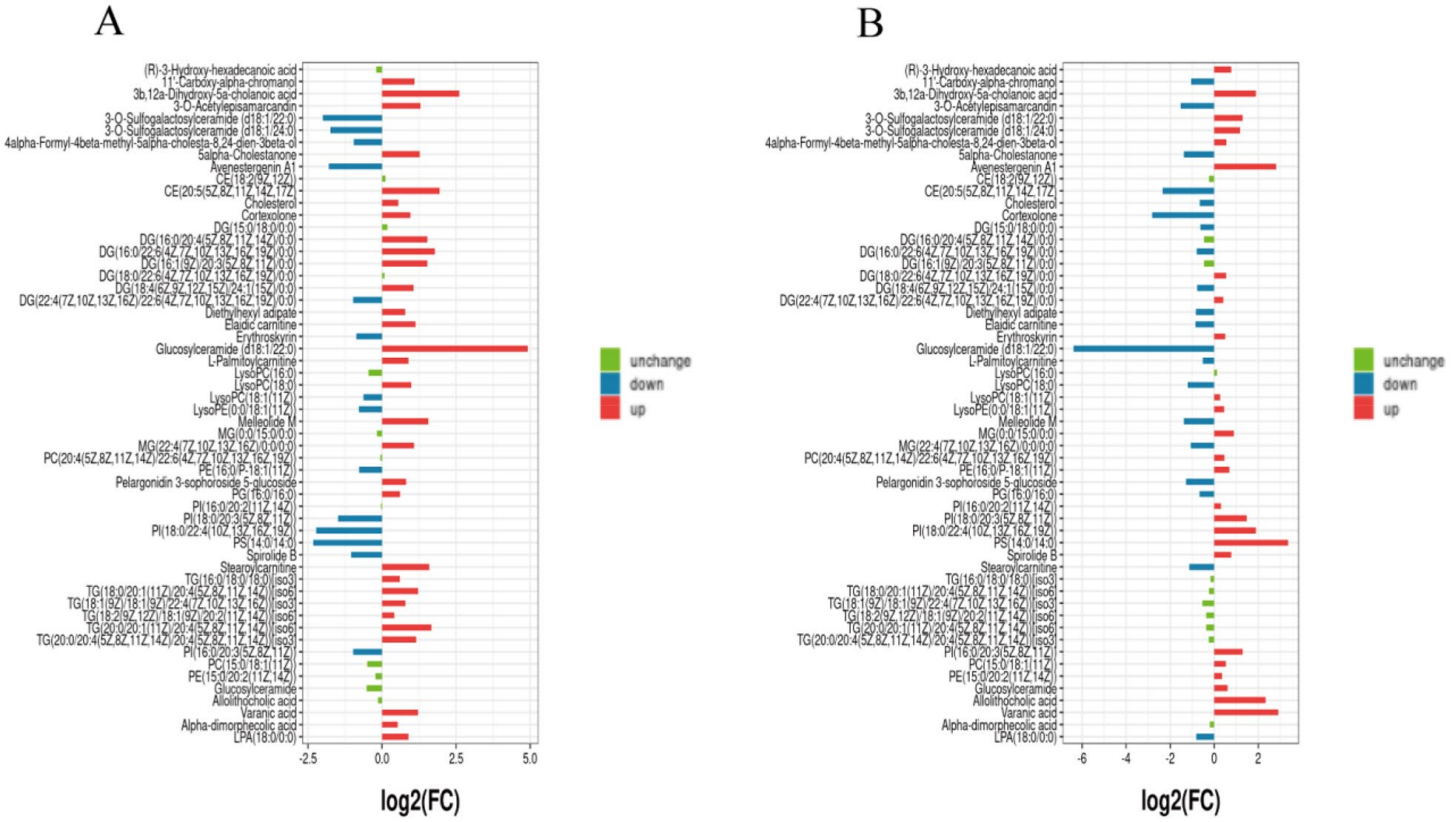
**A** The bar chart of FC values of model and Control groups; **B** the bar chart of FC values of Sch and Model group. (FC value > 1.2 was considered as increased expression, FC value < 0.67 was considered as decreased expression, and the rest were considered as unchanged expression. Green: unchanged; Blue: down; Red: up)

We found a total of 56 significantly variable differential metabolites (Additional file 2: Table S1). The 56 metabolites with significant changes were analyzed by cluster analysis (Fig. 5). We find that there is a disturbance in plasma lipid metabolism, and schisandrin can improve the disorder. In the final screening of differential metabolites, the following situation was found: 1. Cholesterol, CE, TG, DG and 5alpha-cholestanone levels were high in AD model rats, and decreased after treatment with schisandrin; 2. The levels of PC, PI, PE, LysoPC and LysoPE were low in AD model rats, and increased after schisandrin treatment, but it was lower than the Control group. In Fig. 6, FC values of 56 significant differential metabolites were analyzed. We found that Glucosylceramide (d18:1/22:0) increases the maximum in the model/control group and decreases the most in the Sch/Model group. In summary, we found that schisandrin can call back most of the imbalanced lipid metabolites.

### Molecular docking indicates schisandrin interacts with LXRβ

**Fig. 7:**
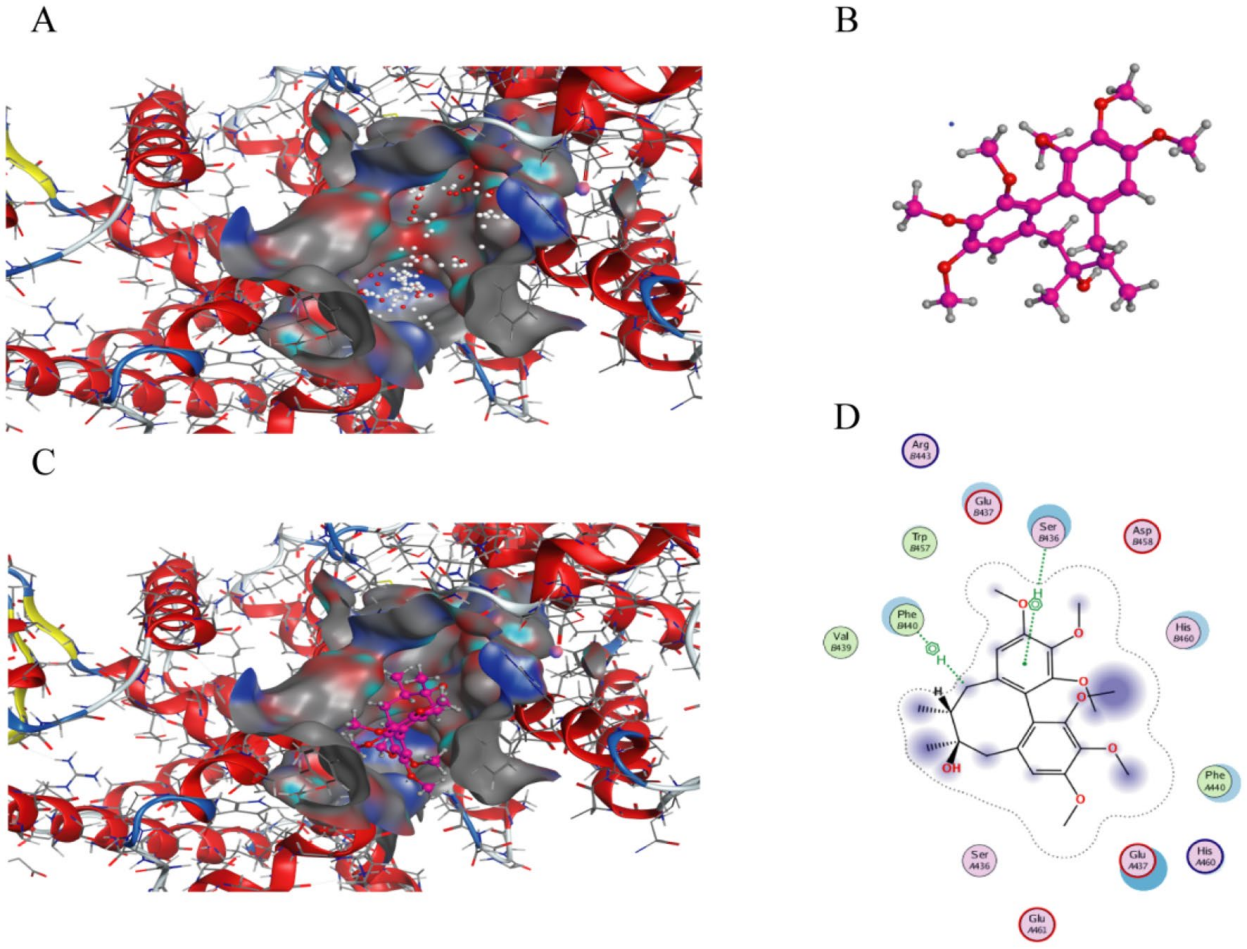
**A** The possible active site is shown in white and red spheres; **B** the receptor goes into the active pocket; **C** the receptor form of the optimal conformation; **D** two-dimensional interaction map of schisandrin and inward-facing structure of LXRβ

Based on molecular docking scores and interactions, the sixth conformation was selected as the dominant conformation. The 3-dimensional (Fig. 7C) and 2-dimensional (Fig. 7D) interaction map of the molecular docking assay showed that the interaction between schisandrin and LXRβ. Schisandrin formed 2 H-bond interactions with SerB436 and PheB440 of LXRβ.

### Schisandrin has a protective effect on AD model cells and reduces their Aβ content

**Fig. 8:**
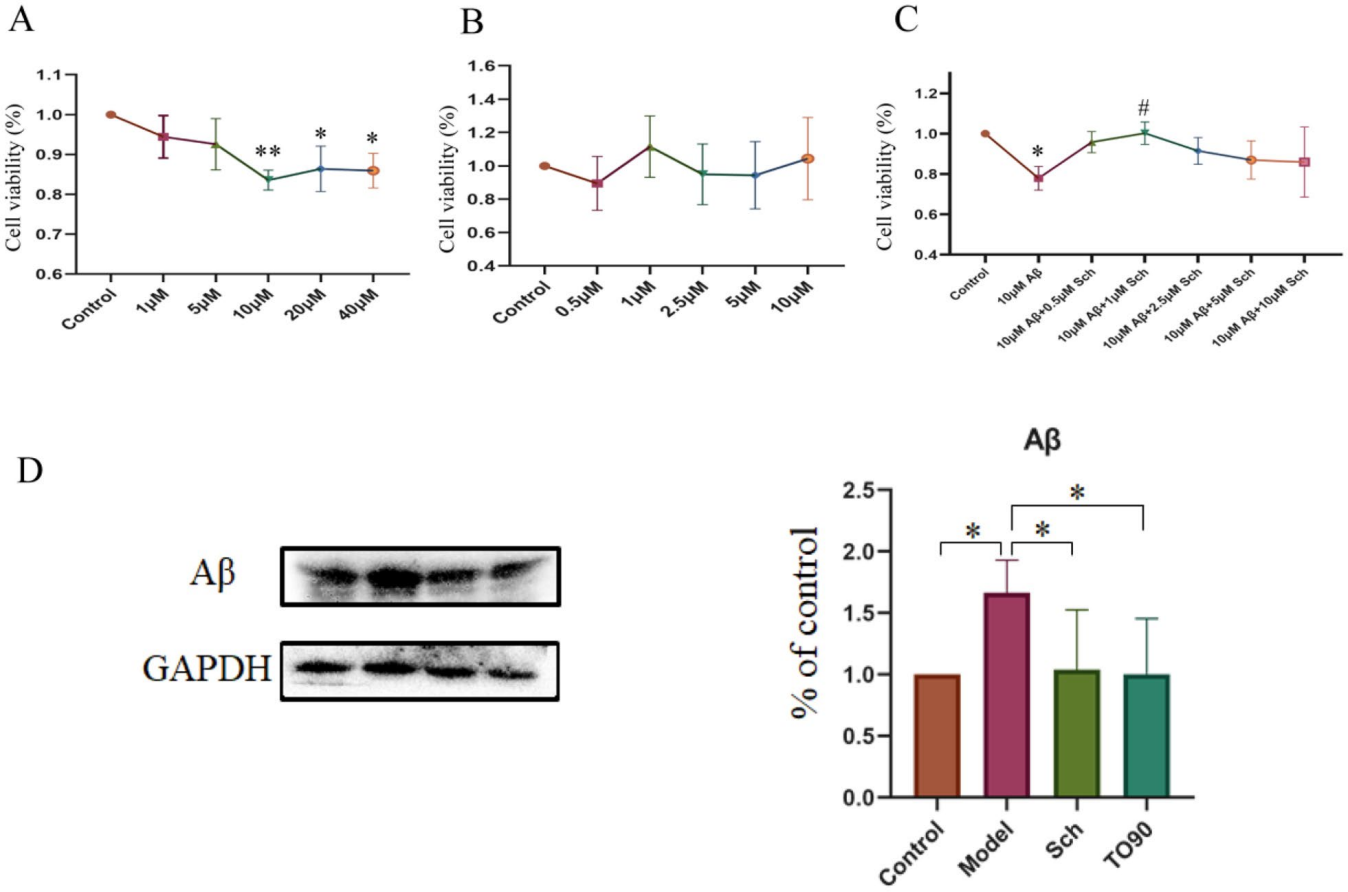
**A** toxicity of Aβ_25-35_ to BV2 cells; **B** toxicity of schisandrin to BV2 cells; **C** protective effect of schisandrin on AD cells; **D** content of Aβ in AD model cells under different treatments. (*p < 0.05, **p < 0.01)

BV2 cells were treated with different concentrations of Aβ_25–35_ for 24 h, and the cell viability was measured by CCK8 method as shown in Fig. 8A. Experimental results showed that the cell viability was the lowest under the action of 10 μM Aβ_25-35_. Combined with literature research, the cells treated with 10 μM Aβ_25–35_ were selected as Model group and used in subsequent experiments. Different concentrations of schisandrin were applied to BV2 cells for 24 h, and the cell viability was measured by CCK8 method as shown in Fig. 8B. Schisandrin with 1 μM was the best to improve the cell viability, so schisandrin with 1 μM was selected for subsequent experiments. The BV2 cells were pretreated with schisandrin of different concentrations for 1 h, and then Aβ_25–35_ was added for modeling. After 24 h culture, cell viability measured by CCK8 method was shown in Fig. 8C. Compared with Model group, schisandrin at 1 μM could significantly improve the viability of model cells. Thus, 10 μM Aβ_25–35_ + 1 μM schisandrin is used as the Sch group, and 10 μM Aβ_25–35_ + 10 μM to901317 is used as the TO90 group. In Fig. 8D, the results of the western blot experiment showed that after the treatment of schisandrin and to901317, the Aβ content in BV2 cells decreased significantly.

### Schisandrin can alleviate apoptosis in AD model cells

**Fig. 9:**
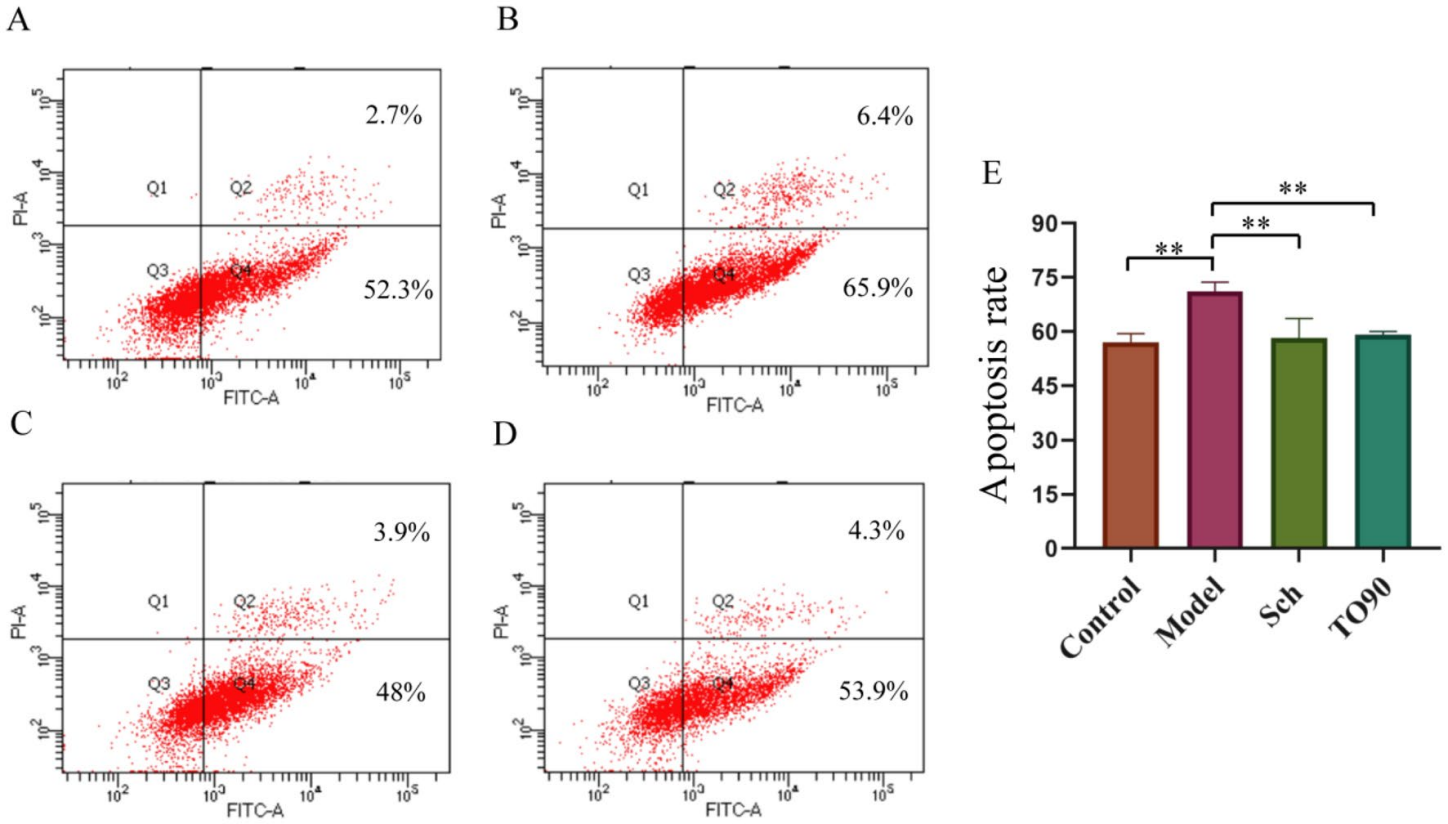
**A** the Control group; **B** the Model group; **C** the Sch group; **D** the TO90 group; **E** apoptosis rates of different groups of cells (**p < 0.01)

Compared with the Control group, the apoptosis rate of cells in the Model group was elevated. However, the apoptosis rate decreased significantly after administration of schisandrin and to901317 treatments but lower than in the Control group. Aβ can induce apoptosis of BV2 cells, while schisandrin and to901317 can inhibit apoptosis of BV2 cells, indicating that schisandrin has a neurotrophic effect (Fig. 9).

### Schisandrin activates LXR and enhances protein and mRNA expression of APOE and ABCA1

**Fig. 10:**
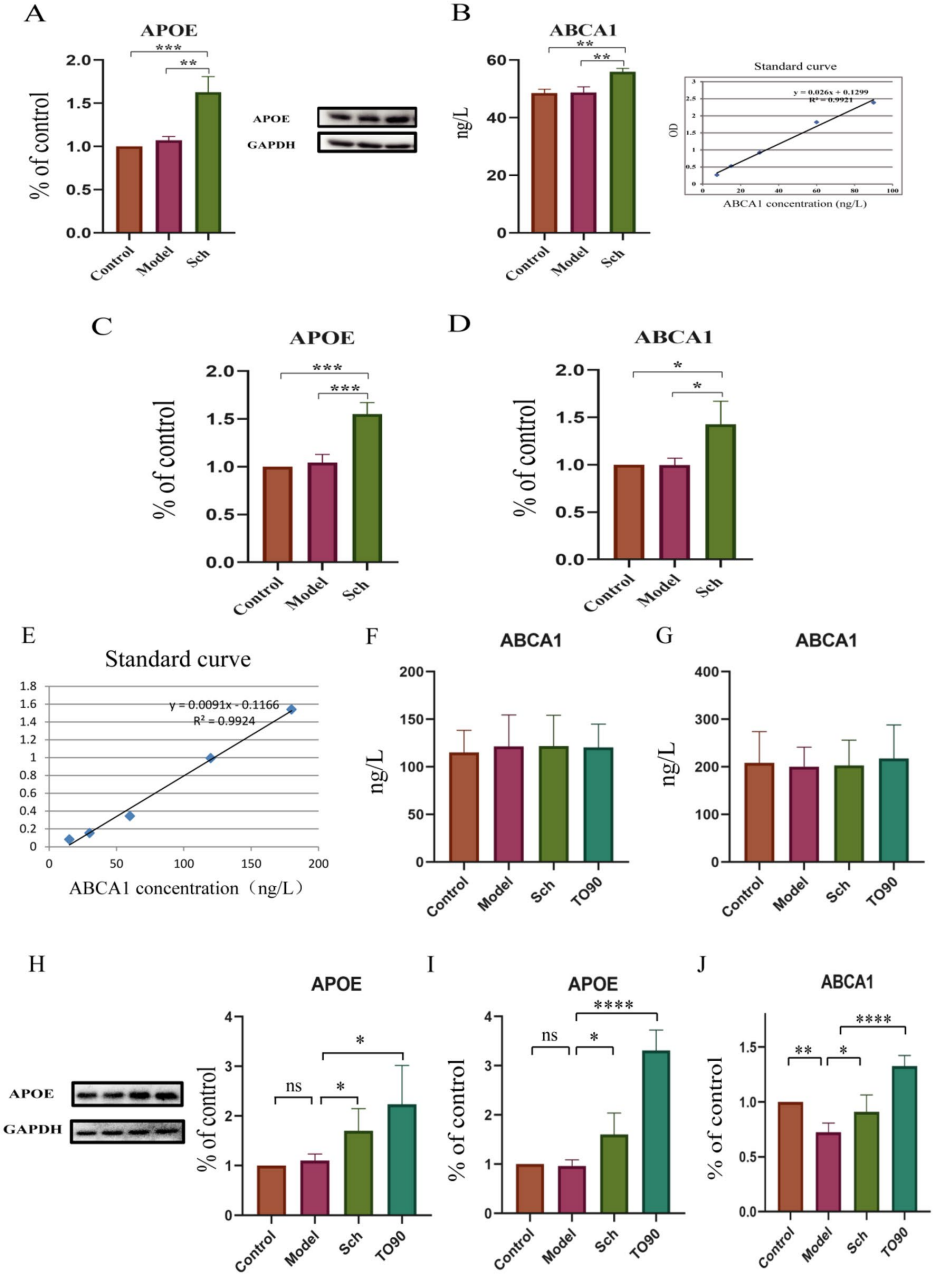
**A**, **B** Expression of APOE and ABCA1 protein in brain tissue; **C**, **D** the mRNA expression of APOE and ABCA1 in brain tissue. **E** Standard curve of cell sample; **F** expression of ABCA1 protein in BV2 cells; **G** expression of ABCA1 protein in BV2 cell culture medium; **H** expression of APOE protein in BV2 cells; **I**, **J** the mRNA expression of APOE and ABCA1 in BV2 cells (*p < 0.05, **p < 0.01, ***p < 0.001, ****p < 0.0001)

In the animal part, we find that although the APOE expression level in the Model group was slightly higher than that in the Control group, the difference was not statistically significant. The mRNA expression of APOE and ABCA1 in Control group and Model group was basically the same, while the mRNA expression of ABCA1 in Sch group was significantly increased. The results showed that schisandrin could promote the expression of APOE and ABCA1 proteins and mRNA in rat brains. In the cell part, we find that no difference in ABCA1 protein expression in BV2 cells (Fig. 10F) or in cell culture medium (Fig. 10G) between groups. However, compared with the Model group, the expression of APOE protein was significantly increased in Sch and TO90 groups. In addition, the mRNA expression of APOE and ABCA1 in Sch and TO90 groups was significantly higher than that in the model group (Fig. 10I–J). We speculate that schisandrin accelerates the clearance of Aβ by activating LXR receptors, promoting the expression of APOE and ABCA1, and thus improving AD pathology.

## Discussion

Hippocampus is the important areas associated with the learning and memory function formation in the mammalian brain. Intracerebroventricular (ICV) injection of Aβ_25-35_ is a universally accepted method to establish the model of AD. In our study, intraventricular injection Aβ_25-35_ caused a significant learning and memory impairments in adult rats which were demonstrated by MWM testing. Our results suggest that schisandrin can improve the learning and memory ability of AD rats. The result of immunohistochemistry showed that the model was reliable. In addition, the content of Aβ in the brain tissue of AD rats treated with schisandrin decreased significantly, indicating that schisandrin can reduce the level of Aβ in the brain tissue of AD rats.

A large number of studies have found that lipid metabolism disorders occur in AD [10]. Since important biochemical changes in the brain can be reflected in the peripheral plasma, we conducted plasma lipidomic studies. Our study found that in peripheral plasma, lipid metabolism disorders in AD rats, while schisandrin can improve that. Our results showed that Cholesterol, CE, TGs, DGs and 5alpha-cholestanone levels were high in AD rats, but decreased after schisandrin treatment. Cholesterol is related to the development of AD. Cholesterol and cholesterol esters play important roles in amyloidogenesis [6]. Low expressions of cholesteryl esters and free cholesterol correlated with an increase of Aβ production and loss of neuronal membrane cholesterol caused amyloidogenesis [1]. TGs are the most predominant glycerolipids. A close relationship between TGs and cognitive impairment has been demonstrated in animal studies, which suggest that lowering triglycerides can reverse cognitive impairment and enhance oxidative stress in the brain [8]. In addition, other studies have found elevated serum DGs levels in AD patients [2]. As previously reported [24], phospholipids such as PC, PI, PE, LysoPC and LysoPE were also found to be low in AD rats in our study. Studies have found that the expression of sphingolipid metabolism enzymes in the AD brain changes, resulting in increased ceramide levels. Our study found that schisandrin lowered levels of Glucosylceramide (d18:1/22:0) in the plasma of AD rats. These results suggest that schisandrin can modulate abnormal lipid metabolism in AD rats.

In the brain, LXR acts as a central regulator of cholesterol [15, 17]. It has been shown that the activation of LXR leads to an increased expression of APOE and ABCA1 (Fan et al., 2018). APOE is a lipid-binding protein responsible for lipid and cholesterol transport and metabolism (Chen et al., 2021). In addition, APOE is responsible for the transport and metabolism of triglycerides and regulates the clearance of cholesterol and triglycerides from plasma (Phillips, 2014). Based on our findings about changes in cholesterol-related lipid metabolism and pathways. We speculate that there may be a link between schisandrin and LXR. LXRa and LXRβ are two subtypes of LXR. Both LXRa and LXRβ are expressed in the mammalian brain and the expression level of LXRβ is 2–5 folds higher than LXRa [21]. LXRa is mainly expressed in organs associated with lipid metabolism, including liver, lung and kidney, while LXRβ is widely expressed in all tissues and organs, especially the brain [20]. LXR are an attractive pharmacological target for regulating brain Aβ levels, but as specific agents for neurodegenerative diseases, agonists should be designed to penetrate the BBB and brain specificity, thereby avoiding systemic distribution, and are therefore preferred for LXRβ isomer specific ligands [22]. Considering that schisandrin has good penetration of the BBB, we further performed molecular docking between schisandrin and LXRβ to preliminarily predict whether there is an interaction. In our study, molecular docking results show that schisandrin interacts with LXRβ.

Our study demonstrated increased protein and mRNA levels of APOE and ABCA1 in AD rats after administration of schisandrin treatment. In addition, we further verified on microglia BV2. Firstly, we found that schisandrin can improve the apoptosis of BV2 microglia and has a protective effect on BV2 microglia. Secondly, the results showed that the APOE protein and mRNA levels of BV2 cells increased after schisandrin treatment. Although the protein expression level of ABCA1 was not different among the groups, the mRNA expression level of ABCA1 was increased by schisandrin treatment compared with Model group. Therefore, based on our results, we suggest that schisandrin may improve pathology in AD rats by activating LXR.

## Conclusions

In this study, we explored the possible mechanisms by which schisandrin might improve AD pathology. Based on plasma lipidomics results, we speculate a possible association to LXR. Based on the molecular docking results, we further hypothesized that schisandrin may act on the LXRβ. We detected LXR downstream target genes APOE and ABCA1 protein and mRNA expression levels on brain tissue and BV2 cells, respectively, after schisandra treatment. As previously envisioned, the protein and mRNA expression of APOE and ABCA1 were increased after the administration of schisandrin treatment. Based on the above experimental results, we conclude that schisandrin may improve the pathology of AD rats by activating LXR.

## Supplementary Information

**Additional file 1.** S-plots in the negative and positive ion modes.** A:** S-plot of Control and Model group; **B:** S-plot of Model and Sch group; **C:** S-plot of Control and Model group; **D:** S-plot of Model and Sch group. (A and Bin positive ion mode, C and D in positive ion mode).

**Additional file 2.** Table of differential metabolites.

## Data Availability

Data and materials available on request from the authors.

## Abbreviations

AD: Alzheimer’s disease
LXR: Liver X Receptor
APOE: Apolipoprotein E
ABCA1: ATP binding cassette transporter A1
BBB: Blood–brain barrier
QC: Quality control
PCA: Principal component analysis
OPLS-DA: Orthogonal partial least squares discriminant analysis
