# Supplementary figures and images for: Combined with UPLC-Triple-TOF/MS-based plasma lipidomics and molecular pharmacology reveals the mechanisms of schisandrin against Alzheimer’s disease
Source: Chin Med. 2023 Feb 6;18:11. doi: 10.1186/s13020-023-00714-y (PMC9903588; doi:10.1186/s13020-023-00714-y)

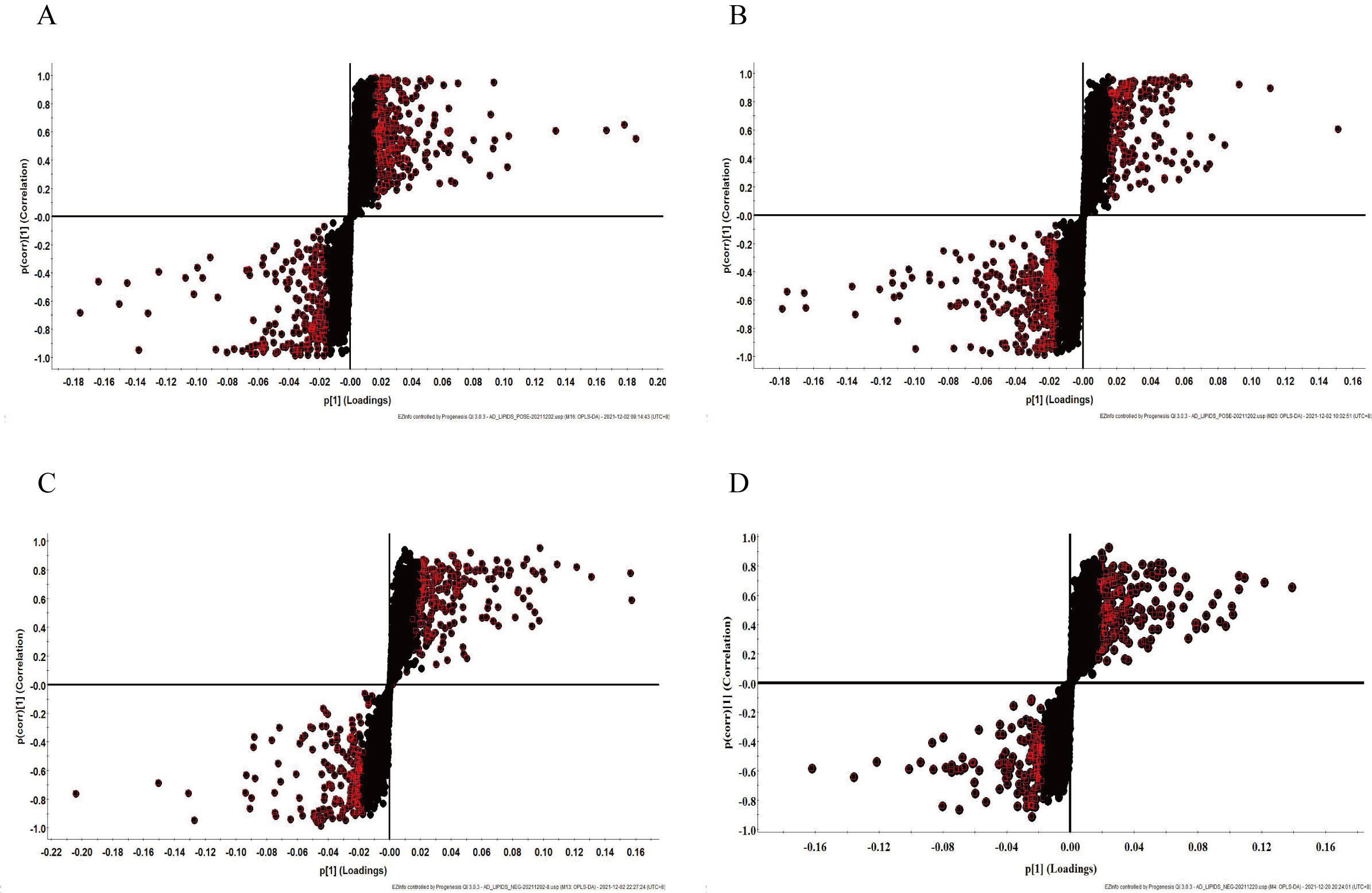

Supplement: Supplementary file 1 — Additional file 1. S-plots in the negative and positive ion modes. A: S-plot of Control and Model group; B: S-plot of Model and Sch group; C: S-plot of Control and Model group; D: S-plot of Model and Sch group. (A and Bin positive ion mode, C and D in positive ion mode). [file 13020_2023_714_MOESM1_ESM.tif]
