# Supplementary material for: Combined with UPLC-Triple-TOF/MS-based plasma lipidomics and molecular pharmacology reveals the mechanisms of schisandrin against Alzheimer’s disease
Source: Chin Med. 2023 Feb 6;18:11. doi: 10.1186/s13020-023-00714-y (PMC9903588; doi:10.1186/s13020-023-00714-y)
Supplement: Supplementary file 2 — Additional file 2. Table of differential metabolites. [file 13020_2023_714_MOESM2_ESM.docx]

Table S1 Table of differential metabolites

| Compund ID | Adducts | Formula | Description | m/z | Retention time(min) | Anova (p) | FC-MC | FC-SM |
| --- | --- | --- | --- | --- | --- | --- | --- | --- |
| HMDB10734 | M+NH4 | C16H32O3 | (R)-3-Hydroxy-hexadecanoic acid | 290.27 | 2.826 | 0.004052177 | 0.87 | 1.7 |
| HMDB12515 | M+H, M+Na | C26H42O4 | 11'-Carboxy-alpha-chromanol | 419.32 | 10.92 | 9.66E-05 | 2.13 | 0.5 |
| HMDB00348 | M+NH4 | C24H40O4 | 3b,12a-Dihydroxy-5a-cholanoic acid | 410.33 | 2.732 | 0.000141707 | 6.1 | 3.7 |
| HMDB31958 | M+NH4 | C26H34O6 | 3-O-Acetylepisamarcandin | 460.27 | 2.357 | 0.000403079 | 2.45 | 0.3 |
| HMDB12316 | M+H | C46H89NO11S | 3-O-Sulfogalactosylceramide (d18:1/22:0) | 864.62 | 12.52 | 7.84E-06 | 0.25 | 2.5 |
| HMDB00024 | M+H | C48H93NO11S | 3-O-Sulfogalactosylceramide (d18:1/24:0) | 892.66 | 13.25 | 8.06E-05 | 0.3 | 2.3 |
| HMDB12167 | M+Na | C29H46O2 | 4alpha-Formyl-4beta-methyl-5alpha-cholesta-8,24-dien-3beta-ol | 449.34 | 10.71 | 1.44E-07 | 0.51 | 1.5 |
| HMDB00871 | M+H-H2O | C27H46O | 5alpha-Cholestanone | 369.35 | 16.17 | 0.000379848 | 2.42 | 0.4 |
| HMDB35264 | M+H-H2O | C38H55NO7 | Avenestergenin A1 | 620.39 | 6.448 | 5.50E-07 | 0.29 | 7 |
| HMDB00610 | M+H, M+NH4 | C45H76O2 | CE(18:2(9Z,12Z)) | 666.62 | 18.3 | 2.85E-05 | 1.08 | 0.8 |
| HMDB06731 | M+H | C47H74O2 | CE(20:5(5Z,8Z,11Z,14Z,17Z) | 671.58 | 16.17 | 0.000156149 | 3.85 | 0.2 |
| HMDB00067 | M+H-H2O | C27H46O | Cholesterol | 369.35 | 16.32 | 2.30E-05 | 1.46 | 0.6 |
| HMDB00015 | M+H | C21H30O4 | Cortexolone | 347.22 | 1.471 | 1.74E-05 | 1.94 | 0.1 |
| HMDB07071 | M+H-H2O, M+NH4 | C36H70O5 | DG(15:0/18:0/0:0) | 565.52 | 14.98 | 4.20E-06 | 1.13 | 0.6 |
| HMDB07112 | M+NH4, M+Na | C39H68O5 | DG(16:0/20:4(5Z,8Z,11Z,14Z)/0:0) | 634.54 | 14.21 | 0.001391883 | 2.87 | 0.7 |
| HMDB07121 | M+NH4, M+Na, M+H | C41H68O5 | DG(16:0/22:6(4Z,7Z,10Z,13Z,16Z,19Z)/0:0) | 658.54 | 14.08 | 0.032004125 | 3.43 | 0.6 |
| HMDB07139 | M+NH4, M+Na | C39H68O5 | DG(16:1(9Z)/20:3(5Z,8Z,11Z)/0:0) | 634.54 | 14.21 | 0.001391883 | 2.87 | 0.7 |
| HMDB07179 | M+H-H2O | C43H72O5 | DG(18:0/22:6(4Z,7Z,10Z,13Z,16Z,19Z)/0:0) | 651.53 | 11.87 | 0.001578375 | 1.05 | 1.5 |
| HMDB07355 | M+H, M+Na | C45H78O5 | DG(18:4(6Z,9Z,12Z,15Z)/24:1(15Z)/0:0) | 699.6 | 15.98 | 5.08E-06 | 2.09 | 0.6 |
| HMDB07701 | M+Na | C47H72O5 | DG(22:4(7Z,10Z,13Z,16Z)/22:6(4Z,7Z,10Z,13Z,16Z,19Z)/0:0) | 739.53 | 12.39 | 1.98E-06 | 0.51 | 1.3 |
| HMDB40270 | M+H | C22H42O4 | Diethylhexyl adipate | 371.32 | 9.963 | 3.41E-07 | 1.72 | 0.6 |
| HMDB06464 | M+H | C25H47NO4 | Elaidic carnitine | 426.36 | 5.978 | 0.028699562 | 2.18 | 0.6 |
| HMDB30464 | M+NH4 | C26H33NO6 | Erythroskyrin | 473.26 | 2.38 | 2.38E-09 | 0.55 | 1.4 |
| HMDB04974 | M+H-H2O | C46H89NO8 | Glucosylceramide (d18:1/22:0) | 766.66 | 14.21 | 0.000619254 | 30.2 | 0 |
| HMDB00222 | M+H, M+Na | C23H45NO4 | L-Palmitoylcarnitine | 400.34 | 5.532 | 0.044176595 | 1.85 | 0.7 |
| HMDB10382 | M+H | C24H50NO7P | LysoPC(16:0) | 496.34 | 5.861 | 0.000585541 | 0.73 | 1.1 |
| HMDB10384 | M+Na | C26H54NO7P | LysoPC(18:0) | 546.35 | 7.349 | 7.69E-07 | 1.98 | 0.4 |
| HMDB10385 | M+H-H2O, M+H | C26H52NO7P | LysoPC(18:1(11Z)) | 522.36 | 6.25 | 0.001198175 | 0.65 | 1.2 |
| HMDB11475 | M+H | C23H46NO7P | LysoPE(0:0/18:1(11Z)) | 480.31 | 6.401 | 0.000172163 | 0.58 | 1.4 |
| HMDB35068 | M+H | C23H29ClO7 | Melleolide M | 453.17 | 2.79 | 0.000537876 | 2.96 | 0.4 |
| HMDB11532 | M+NH4 | C18H36O4 | MG(0:0/15:0/0:0) | 334.29 | 2.755 | 0.004180502 | 0.88 | 1.8 |
| HMDB11584 | M+NH4 | C25H42O4 | MG(22:4(7Z,10Z,13Z,16Z)/0:0/0:0) | 424.34 | 4.878 | 0.000574653 | 2.11 | 0.5 |
| HMDB08452 | M+H | C50H80NO8P | PC(20:4(5Z,8Z,11Z,14Z)/22:6(4Z,7Z,10Z,13Z,16Z,19Z)) | 854.57 | 11.97 | 0.000180817 | 0.96 | 1.4 |
| HMDB08951 | M+NH4 | C39H76NO7P | PE(16:0/P-18:1(11Z)) | 719.57 | 11.95 | 0.000162373 | 0.59 | 1.6 |
| HMDB33687 | M+H | C33H41O20+ | Pelargonidin 3-sophoroside 5-glucoside | 758.22 | 15.32 | 5.77E-06 | 1.76 | 0.4 |
| HMDB10570 | M+NH4 | C38H75O10P | PG(16:0/16:0) | 740.55 | 11.48 | 9.82E-05 | 1.52 | 0.6 |
| HMDB09786 | M+H, M+NH4 | C45H83O13P | PI(16:0/20:2(11Z,14Z)) | 880.59 | 12.04 | 0.032498654 | 0.97 | 1.2 |
| HMDB09813 | M+H, M+Na, M+NH4 | C47H85O13P | PI(18:0/20:3(5Z,8Z,11Z)) | 906.61 | 12.42 | 0.012034983 | 0.36 | 2.8 |
| HMDB09817 | M+NH4, M+Na | C49H87O13P | PI(18:0/22:4(10Z,13Z,16Z,19Z)) | 932.63 | 10.84 | 7.76E-05 | 0.21 | 3.7 |
| HMDB12330 | M+H-H2O | C34H66NO10P | PS(14:0/14:0) | 662.44 | 8.103 | 1.61E-06 | 0.2 | 10 |
| HMDB30492 | M+NH4 | C42H63NO7 | Spirolide B | 711.49 | 11.48 | 0.000491747 | 0.49 | 1.7 |
| HMDB00848 | M+H | C25H49NO4 | Stearoylcarnitine | 428.37 | 7.196 | 0.000590013 | 3.01 | 0.5 |
| HMDB05365 | M+NH4, M+Na | C55H106O6 | TG(16:0/18:0/18:0)[iso3] | 880.83 | 18.46 | 0.00027138 | 1.51 | 0.9 |
| HMDB05408 | M+NH4, M+Na | C61H108O6 | TG(18:0/20:1(11Z)/20:4(5Z,8Z,11Z,14Z))[iso6] | 954.85 | 18.26 | 0.000483823 | 2.33 | 0.8 |
| HMDB10467 | M+NH4 | C61H106O6 | TG(18:1(9Z)/18:1(9Z)/22:4(7Z,10Z,13Z,16Z))[iso3] | 952.83 | 18 | 0.001895015 | 1.73 | 0.7 |
| HMDB10484 | M+NH4, M+Na | C59H104O6 | TG(18:2(9Z,12Z)/18:1(9Z)/20:2(11Z,14Z))[iso6] | 926.82 | 17.9 | 0.000391234 | 1.33 | 0.8 |
| HMDB05419 | M+Na | C63H112O6 | TG(20:0/20:1(11Z)/20:4(5Z,8Z,11Z,14Z))[iso6] | 987.84 | 18.52 | 0.026228223 | 3.16 | 0.8 |
| HMDB05420 | M+NH4 | C63H106O6 | TG(20:0/20:4(5Z,8Z,11Z,14Z)/20:4(5Z,8Z,11Z,14Z))[iso3] | 976.83 | 17.91 | 0.002311178 | 2.22 | 0.8 |
| HMDB09787 | M-H | C45H81O13P | PI(16:0/20:3(5Z,8Z,11Z)) | 859.53 | 11.69 | 0.009805812 | 0.51 | 2.4 |
| HMDB07938 | M-H | C41H80NO8P | PC(15:0/18:1(11Z)) | 744.55 | 13.16 | 0.045314323 | 0.71 | 1.4 |
| HMDB08901 | M-H, M+Cl | C40H76NO8P | PE(15:0/20:2(11Z,14Z)) | 764.55 | 12.28 | 0.017123396 | 0.86 | 1.3 |
| HMDB00140 | M+Cl | C48H93NO8 | Glucosylceramide | 846.66 | 15.13 | 0.002535324 | 0.7 | 1.5 |
| HMDB00381 | M-H | C24H40O3 | Allolithocholic acid | 375.29 | 3.949 | 5.20E-05 | 0.91 | 5 |
| HMDB02195 | M-H, M+Cl, M+Na-2H | C26H44O5 | Varanic acid | 435.31 | 3.949 | 0.000679714 | 2.33 | 7.5 |
| HMDB04670 | M-H | C18H32O3 | Alpha-dimorphecolic acid | 295.23 | 3.353 | 0.037237847 | 1.45 | 0.9 |
| HMDB07854 | M-H | C21H43O7P | LPA(18:0/0:0) | 437.27 | 7.513 | 0.015897506 | 1.86 | 0.6 |
